# Short-term outcome of Ivor Lewis esophagectomy following neoadjuvant chemoradiation versus perioperative chemotherapy in patients with locally advanced adenocarcinoma of the esophagus and gastroesophageal junction: a propensity score-matched analysis

**DOI:** 10.1007/s00432-021-03720-5

**Published:** 2021-07-05

**Authors:** Patrick Sven Plum, Alexander Damanakis, Lisa Buschmann, Angela Ernst, Rabi Raj Datta, Lars Mortimer Schiffmann, Thomas Zander, Hans Fuchs, Seung-Hun Chon, Hakan Alakus, Wolfgang Schröder, Arnulf Heinrich Hölscher, Christiane Josephine Bruns, Marc Bludau

**Affiliations:** 1 Department of General, Visceral, Cancer and Transplantation Surgery, Faculty of Medicine and University Hospital Cologne, University of Cologne, Kerpener Straße 62, 50937 Cologne, Germany; 2 Department of Pediatrics, Klinikum Konstanz, 78464 Constance, Germany; 3 Institute of Medical Statistics and Computational Biology, Faculty of Medicine and University Hospital Cologne, University of Cologne, Kerpener Straße 62, 50937 Cologne, Germany; 4 Department I of Internal Medicine, Faculty of Medicine and University Hospital Cologne, University of Cologne, Kerpener Straße 62, 50937 Cologne, Germany; 5 Center for Esophageal Diseases, Elisabeth-Krankenhaus Essen, Klara-Kopp-Weg 1, 45138 Essen, Germany; 6 Else Kröner Forschungskolleg Cologne “Clonal Evolution in Cancer”, Faculty of Medicine and University Hospital, Weyertal 115B, Cologne, 50937 Germany

**Keywords:** Esophageal/gastroesophageal adenocarcinoma, Chemoradiation, Chemotherapy, Neoadjuvant treatment, Ivor Lewis esophagectomy, Outcome, Prognosis

## Abstract

**Background:**

Patients with locally advanced esophageal or gastroesophageal adenocarcinoma benefit from multimodal therapy concepts including neoadjuvant chemoradiation (nCRT), respectively, perioperative chemotherapy (pCT). However, it remains unclear which treatment is superior concerning postoperative morbidity.

**Methods:**

In this study, we compared the postsurgical survival (30-day/90-day/1-year mortality) (primary endpoint), treatment response, and surgical complications (secondary endpoints) of patients who either received nCRT (CROSS protocol) or pCT (FLOT protocol) due to esophageal/gastroesophageal adenocarcinoma. Between January 2013 and December 2017, 873 patients underwent Ivor Lewis esophagectomy in our high-volume center. 339 patients received nCRT and 97 underwent pCT. After 1:1 propensity score matching (matching criteria: sex, age, BMI, ASA score, and Charlson score), 97 patients per subgroup were included for analysis.

**Results:**

After matching, tumor response (ypT/ypN) did not differ significantly between nCRT and pCT (*p* = 0.118, respectively, *p* = 0.174). Residual nodal metastasis occurred more often after pCT (*p* = 0.001). Postsurgical mortality was comparable within both groups. No patient died within 30 or 90 days after surgery while the 1-year survival rate was 72.2% for nCRT and 68.0% for pCT (*p* = 0.47). Only grade 3a complications according to Clavien–Dindo were increased after pCT (*p* = 0.04). There was a trend towards a higher rate of pylorospasm within the pCT group (nCRT: 23.7% versus pCT: 37.1%) (*p* = 0.061). Multivariate analysis identified pCT, younger age, and Charlson score as independent variables for pylorospasm.

**Conclusion:**

Both nCRT and pCT are safe and efficient within the multimodal treatment of esophageal/gastroesophageal adenocarcinoma. We did not observe differences in postoperative morbidity. However, functional aspects such as gastric emptying might be more frequent after pCT.

## Introduction

Adenocarcinoma of the esophageal (EAC) and the gastroesophageal junction are still devastating diseases with only a poor prognosis. The 5-year overall survival rate is about 20% considering all stages of neoplasia (Coleman et al. 2018) while its incidence is supposed to increase within the future (Arnold et al. 2017). The majority of patients is typically diagnosed in an advanced stage and, therefore, benefit from multimodal therapy instead of surgery alone. Nowadays, different treatment concepts coexist: On the one hand, there is the wide-spread neoadjuvant chemoradiation (nCRT) (including a cumulative radiation dose of 41.4 Gy (23 fractions with 1.8 Gy) plus carboplatin/paclitaxel) which was systematically examined within the so-called CROSS trial demonstrating an improved survival among EAC patients (43 months compared to 27 months) (Van Hagen et al. 2012; Shapiro et al. 2015). On the other hand, the common perioperative chemotherapy (pCT) with 5-fluorouracil (5-FU), leucovorin, oxaliplatin, and docetaxel (so-called FLOT protocol) achieved a median overall survival of 50 months in EAC patients (Al-Batran et al. 2019). So far, it remains unclear which concept (chemoradiation or chemotherapy) is superior while both treatment regimens have different adverse side effects: about 12% of patients with nCRT develop esophagitis, thrombocytopenia, neutropenia, or leucopenia (Van Hagen et al. 2012). Patients under pCT show infections, neuropathy, neutropenia, or nausea in 7% of cases (Al-Batran et al. 2019). Retrospective analyses could not identify significant differences considering the patients’ prognosis (Liu et al. 2017; Petrelli et al. 2019). However, first, multicentric prospective studies like the ESOPEC, the Neo-AEGIS, or the TOPGEAR study have been initiated to further validate this issue (Leong et al. 2015; Hoeppner et al. 2016; Reynolds et al. 2017).

ESOPEC (NCT02509286), enrolled in 2016, is a German multicenter randomized phase III trial at 31 study sites comparing the perioperative FLOT regimen to the nCRT according to the CROSS protocol for both, adenocarcinomas of the distal esophagus (EAC) as well as the gastroesophageal junction (GEJ I–III). The trial recruited 438 patients so far (Hoeppner et al. 2016). Neo-AEGIS (NCT01726452) is an international multicenter phase III trial with participating centers in the UK, Denmark, and Ireland which compares the outcome of EAC or GEJ patients after CROSS compared to a modified MAGIC regimen (ECF/ECX or EOF/EOX). Initiated in 2014, it aims to include 540 patients (Reynolds et al. 2017). Since 2009, the TOGEAR study (NCT01924819), a two-armed randomized multicenter trial, has recruited patients with gastric adenocarcinoma or GEJ II–III tumors in 61 sites. The recruitment aim is a total of 752 patients. The trial compares perioperative chemoradiation (including two cycles ECF, then radiation (45 Gy) and 5-FU and three cycles ECF/ECX postsurgical) versus perioperative ECF/ECX chemotherapy (with three cycles before and after surgical resection) (Leong et al. 2015).

As data availability is still very limited, we aimed to retrospectively analyze patients with EAC or GEJ tumors who underwent multimodal therapy with either nCRT or pCT within our surgical high-volume center for oncological surgery of the upper gastrointestinal tract. The primary endpoint was the postsurgical survival subdivided into 30-day mortality, 90-day mortality, and 1-year mortality. Secondary endpoints included treatment response as well as postsurgical in-hospital complications.

## Methods

### Patients

Between January 2013 and December 2017, 873 patients underwent esophagectomy due to cancer of the esophagus or the esophagogastric junction at the Department of General, Visceral, Cancer and Transplantation Surgery, University Hospital of Cologne. The primary staging consisted of esophagogastroduodenoscopy with biopsy, endoscopic ultrasound, and spiral contrast-enhanced computer tomography of thorax and abdomen. All patients were discussed in a multidisciplinary tumor conference to determine the treatment procedures. Patients whose histological subtype was not adenocarcinoma were excluded. Only patients with cT2-4 tumors and/or cN + were considered in the analysis since they qualified for multimodal treatment before surgical resection. In these situations, either neoadjuvant chemoradiation (nCRT) consisting of 41.4 Gy radiation, carboplatin, and paclitaxel (CROSS regimen) or perioperative chemotherapy (pCT) including 5-fluorouracil/leucovorin, oxaliplatin, and docetaxel (FLOT regimen) were performed. Other chemotherapeutic regimens were excluded to avoid a heterogeneous pCT cohort (2013–2016: *n* = 38). Follow-up data of all patients were collected during regular postsurgical visits at the department's outpatient clinics. Data were processed considering the criteria of the Esophagectomy Complication Consensus Group (ECCG) (Low et al. 2015, 2019). This retrospective study was performed in accordance with the guidelines of the Institutional Ethics Committee of the University Hospital of Cologne.

### Surgery

The standard surgical procedure was Ivor Lewis laparoscopic gastrolysis and right transthoracic en bloc esophagectomy including two-field lymphadenectomy of the mediastinal and abdominal lymph nodes. As described before, high intrathoracic esophagogastrostomy was performed via a circular stapler device and all lymph nodes from the esophageal specimens were resected according to a standardized protocol for further histopathological examination (Hölscher et al. 2011; Plum et al. 2018). Postoperative complications were graded according to the Clavien–Dindo classification (Dindo et al. 2004).

### Histopathological work-up

All resected lymph nodes and esophageal specimens were fixed within 5% formaldehyde and embedded in paraffin. Blocks were cut into 5 µm thick slides and samples were stained with hematoxylin and eosin. Histopathological analysis and classification were performed by experienced gastrointestinal pathologists according to the seventh edition of the Union for International Cancer Control/TNM-classification of malignant tumors including tumor localization, depth of tumor infiltration, grading, residual tumor as well as the total number of resected and infiltrated lymph nodes (Sobin et al. 2009).

### Statistical analysis

To account for the non-randomly performed treatment assignment, we performed a 1:1 propensity score matching with a pre-specified caliper of 0.2 to select a fitting control group of patients treated according to the CROSS protocol. Matching was performed according to the following parameters: sex, age, BMI, ASA score, and Charlson score. Quantitative variables were summarized using mean (range) and compared using the *t* test. Qualitative variables were summarized by counts, percentages, and compared using *χ*^2^ and, in the case of ordered alternatives, the Jonckheere–Terpstra test. We performed univariate and multivariable (multinomial) conditional logistic regression analyses concerning the matched design. In the multivariable case, we applied both forward- and backward-regression analysis selecting the best fitting model according to the Akaike information criterion (AIC). Survival curves were plotted using the Kaplan–Meier method and analyzed using the log-rank test. In all analyses, a two-sided *p* < 0.05 was considered statistically significant.

Data were analyzed using SPSS Statistics Version 25 (IBM Corp., Armonk, NY, USA) for Windows (Microsoft Corp, Redmond, WA) and R version 3.3.0.

## Results

### Patient cohort and matching process

**Fig. 1:**
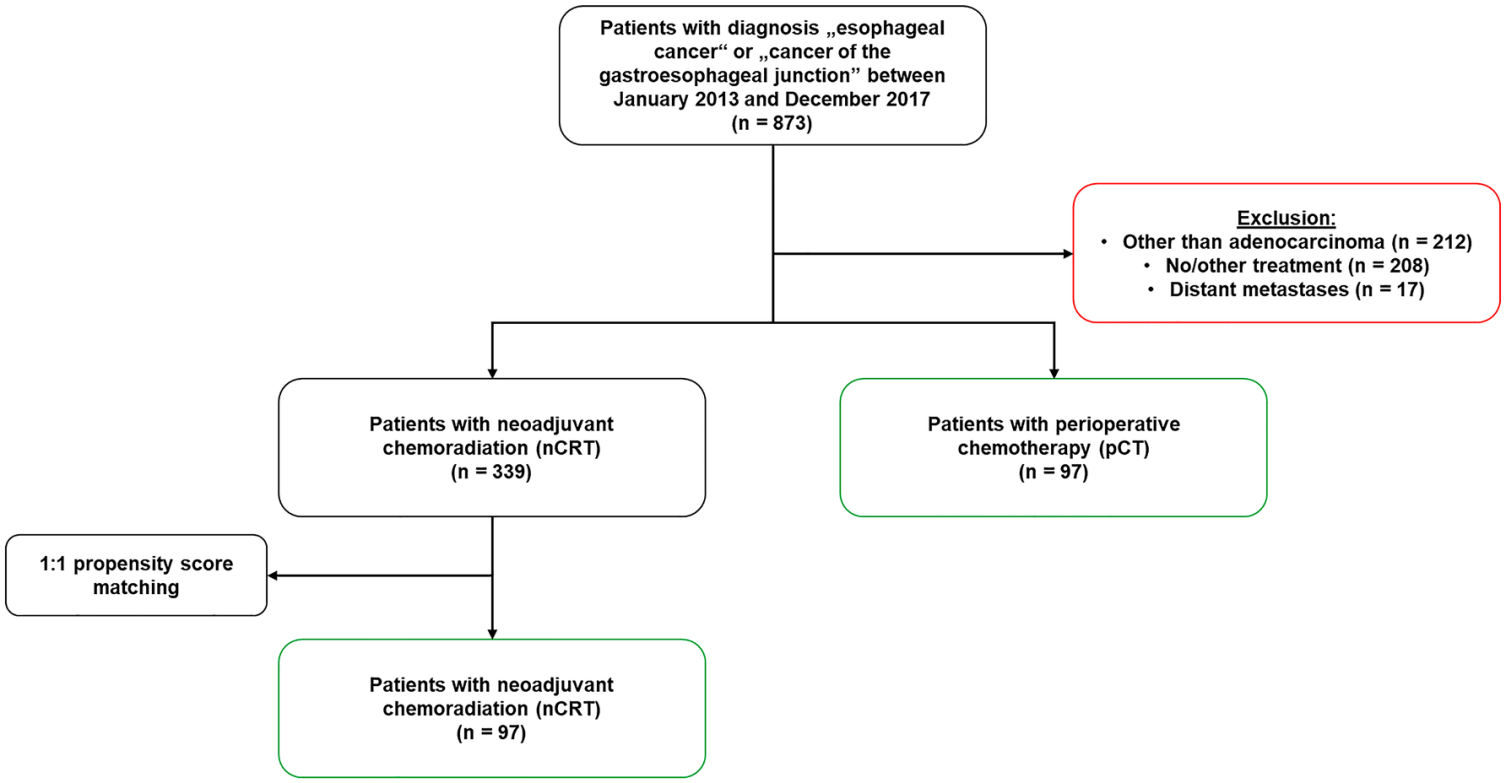
Flow-chart of patient selection and matching

A total of 873 patients with esophageal cancer treated at our center were identified between January 2013 and December 2017 in our prospectively maintained database. We excluded 437 patients from further analysis due to disseminated metastasis at the time of diagnosis (*n* = 17), different histopathological subtype other than adenocarcinoma (*n* = 208), or since they did not qualify for multimodal neoadjuvant treatment (*n* = 212) (including those patients in bad functional conditions as well as patients with only early tumor stages). After preselection, 436 patients remained for the current study of whom 339 patients received nCRT and 97 underwent pCT. A 1:1 propensity score matching of those nCRT patients was performed according to the criteria sex, age, BMI, ASA score, and Charlson score. Figure 1 illustrates the data processing. After matching, we examined the balance of all observed covariates, interactions among all covariates, and quadratic terms of all covariates. Nearly no imbalances remained as assessed through univariate and multivariable tests. Table 1 presents the baseline demographics and initial histopathological results as well as the results after propensity score matching of the patients with nCRT in comparison to those of the pCT group illustrating the process of homogenization.

**Table 1:** Demographic characteristics and the initial histopathological results of neoadjuvant treatment concepts [neoadjuvant chemoradiation (nCRT) versus perioperative chemotherapy (pCT)] comparing all patients with nCRT (*n* = 339) and after propensity score matching (*n* = 97) with the group of patients after pCT (*n* = 97)

Before 1:1 propensity score matching				After 1:1 propensity score matching			
Variable	nCRT	pCT	*p* value	Variable	nCRT	pCT	*p* value
All (*N*; Row—%)	**339 (77.8)**	**97 (22.2)**		All (*N*; Row—%)	**97 (50)**	**97 (50)**	
Age *N*: mean (Std.)	**339: 61.9 (9.68)**	**97: 62.0 (10.87)**	p* = 0.497	Age *N*: mean (Std.)	**97: 62.2 (8.71)**	**97: 62.0 (10.87)**	p* = 0.176
Gender (*N*; Col.—%)			***p***^**†**^** = 0.612**	Gender (*N*; Col.—%)			***p***^**†**^** = 0.663**
Female	47 (13.9)	11 (11.3)		Female	13 (13.4)	11 (11.3)	
Male	292 (86.1)	86 (88.7)		Male	84 (86.6)	86 (88.7)	
BMI *N*: mean (Std.)	**339: 25.9 (4.33)**	**97: 27.6 (4.81)**	p* = 0.197	BMI *N*: mean (Std.)	**97: 27.6 (4.50)**	**97: 27.6 (4.81)**	p* = 0.464
Nodal metastasis *N*: mean (Std.)	**302: 1.67 (3.93)**	**81: 3.31 (5.57)**	p* < 0.001	Nodal metastasis *N*: mean (Std.)	**87: 1.76 (3.30)**	**81: 3.31 (5.57)**	p* = 0.001
Charlson score *N*: mean (Std.)	**339: 3.86 (1.16)**	**97: 3.92 (1.20)**	p* = 0.938	Charlson score *N*: mean	**97: 3.97 (1.13)**	**97: 3.92 (1.20)**	p* = 0.805
Cologne regression scale (*N*; Col.—%)			p** = 0.006	Cologne regression scale (*N*; Col.—%)			p** = 0.464
1	47 (13.9)	25 (31.6)	*p*^†^ = 0.020	1	18 (22.8)	25 (31.6)	
2	82 (242)	23 (29.1)		2	29 (36.7)	23 (29.1)	
3	79 (23.3)	16 (20.3)		3	16 (20.3)	16 (20.3)	
4	75 (22.1)	15 (19.0)		4	16 (20.3)	15 (19.0)	
Missing	56 (16.5)	18 (18.6)		Missing	18 (18.6)	18 (18.6)	
ASA score (*N*; Col.—%)			***p***^**†**^** = 0.344**	ASA score (*N*; Col.**—**%)			p** = 0.845
0	1 (0.3)	0 (0)	p** = 0.167	0	0 (0)	0 (0)	
1	13 (3.8)	5 (5.2)		1	4 (4.1)	5 (5.2)	
2	189 (55.8)	44 (45.4)		2	47 (48.5)	44 (45.4)	
3	134 (39.5)	48 (49.5)		3	46 (47.4)	48 (49.5)	
4	2 (0.6)	0 (0)		4	0 (0)	0 (0)	
pT category (*N*; Col.—%)			***p***^**†**^** = 0.003**	pT category (*N*; Col.**—**%)			***p***^**†**^** = 0.118**
pT0/pTis	80 (23.6)	13 (13.4)	p** = 0.009	pT0/pTis	17 (17.5)	13 (13.4)	p** = 0.356
pT1	60 (17.7)	18 (18.6)		pT1	16 (16.5)	18 (18.6)	
pT2	52 (15.3)	13 (13.4)		pT2	14 (14.4)	13 (13.4)	
pT3	143 (42.2)	46 (47.4)		pT3	49 (50.5)	46 (47.4)	
pT4	3 (0.9)	7 (7.2)		pT4	0 (0)	7 (7.2)	
pTx	1 (0.3)	0 (0)		pTx	1 (1.0)	0 (0)	
pN category (*N*; Col.—%)			***p***^**†**^** = 0.002**	pN category (*N*; Col.**—**%)			***p***^**†**^** = 0.174**
pN0	209 (61.7)	40 (41.2)	p** < 0.001	pN0	55 (56.7)	40 (41.2)	
pN1	57 (16.8)	26 (26.8)		pN1	21 (21.6)	26 (26.8)	
pN2	48 (14.2)	16 (16.5)		pN2	12 (12.4)	16 (16.5)	
pN3	25 (7.4)	15 (15.5)		pN3	9 (9.3)	15 (15.5)	
Grading (*N*; Col.—%)			***p***^**†**^** = 0.626**	Grading (*N*; Col.**—**%)			***p***^**†**^** = 0.997**
0	8 (2.4)	3 (3.1)	p** = 0.411	0	3 (3.1)	3 (3.1)	
G2	56 (16.5)	13 (13.4)		G2	14 (14.4)	13 (13.4)	
G3	74 (21.8)	14 (14.4)		G3	15 (15.5)	14 (14.4)	
Missing	201 (59.3)	67 (69.1)		Missing	65 (67.0)	67 (69.1)	

*t test, **Jonckheere–Terpstra test, ^†^Pearson *χ*^2^ test

*p* < 0.05 was considered statistically significant

### Patient demographics and histopathological results

The demographics of all patients are summarized in Table 1. There were no significant differences according to age, gender, or BMI. Functional parameters such as ASA or Charlson score of those patients were comparable. Histomorphological findings considering the depth of tumor infiltration or the nodal status of the patients did not differ between nCRT and pCT group. The same was true for grading. However, there was a significant difference regarding the number of pathological lymph nodes within the surgical specimens in both groups: among those nCRT patients after matching, a total of 87 nodal metastasis (mean: 1.76) were identified compared to a total of 81 positive lymph nodes (mean: 3.31) within the pCT patients (*p* = 0.001). The same was true for the mean number of retrieved lymph nodes per patient in favor of pCT resulting in a mean of 35.81 harvested lymph nodes per pCT patient versus 28.86 resected lymph nodes per nCRT patient (*p* < 0.001).

The degree of histomorphological regression was classified into four categories (according to the Cologne Regression Scale): grade I > 50% vital residual tumor cells, grade II 10–50% vital residual tumor cells, grade III nearly complete response with < 10% vital residual tumor cells and grade IV complete response (Schneider et al. 2005). There was no difference between both groups. Only a small subgroup of patients in both cohorts achieved a nearly or even a complete response while most patients still had residual tumor cells detectable within their surgical specimens after neoadjuvant treatment.

### Postsurgical complications and prognosis

**Fig. 2:**
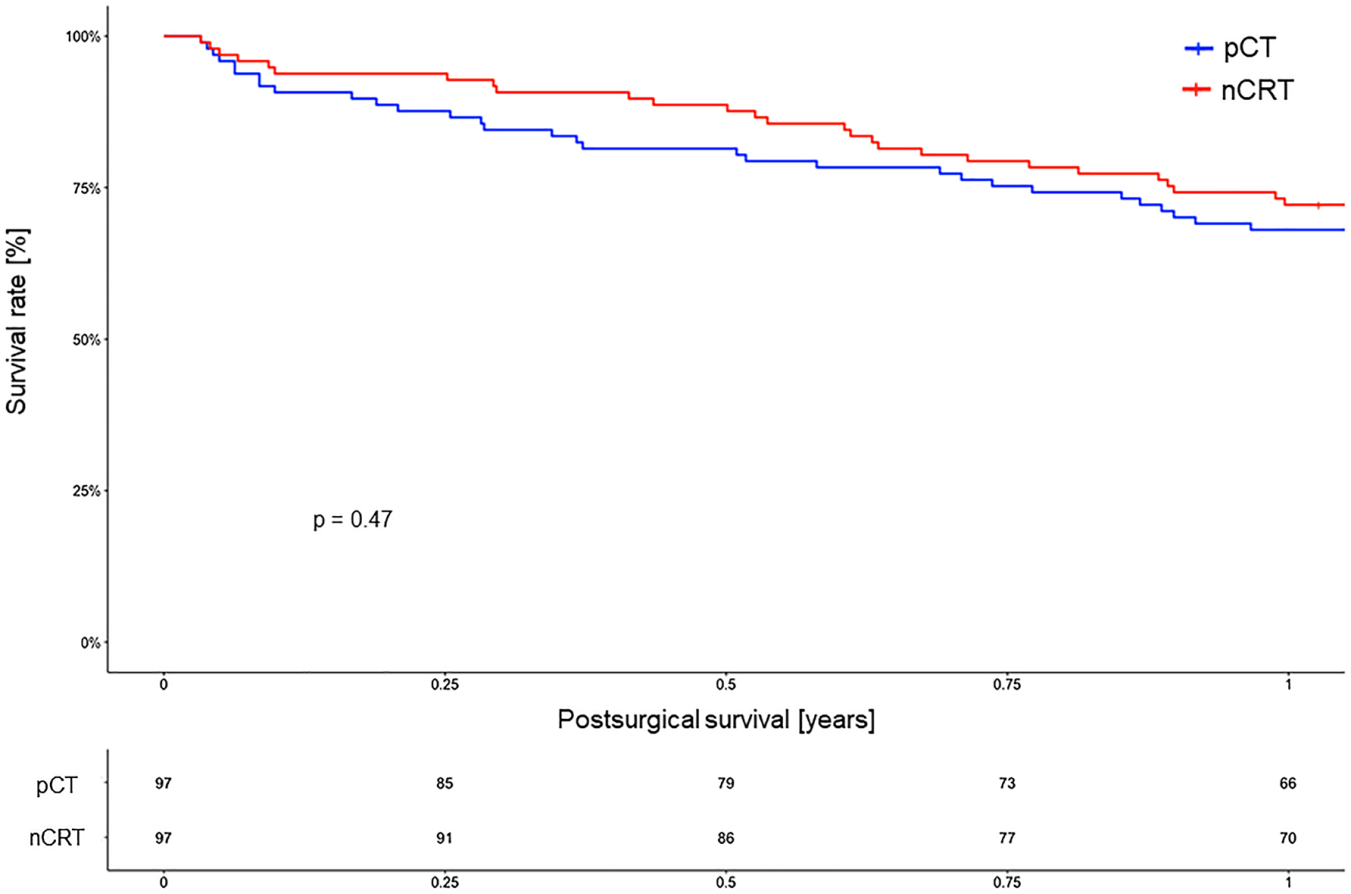
Kaplan–Meier survival analysis (log-rank test) of the 1-year survival for patients with neoadjuvant chemoradiation (nCRT) versus perioperative chemotherapy (pCT)

We performed a univariate analysis for postsurgical follow-up and the occurrence of putative complications as summarized in Table 2. We found no significant differences between both groups, except for complications grade 3a according to the Clavien–Dindo classification (Dindo et al. 2004). There were more such complications (*n* = 42) in the pCT compared to the nCRT cohort (*n* = 30; *p* = 0.04). However, serious complications were equally distributed in both groups. The situation was similar regarding infectious complications (such as pneumonia, wound infection, urinary tract infections, etc.), with no statistically significant differences. Cardiovascular complications such as arrhythmia were found in both cohorts and in both groups only a few patients (*n* = 4 in each group) had to be transferred back to the intensive care unit (ICU). There was a trend towards a more frequent pylorospasm among pCT (*n* = 36) in comparison to nCRT patients (*n* = 23), however, this was non-significant (*p* = 0.061). Prognostically, the postsurgical survival [including the 30-day (no patient died), 90-day (no patient died), and 1-year survival] did not differ between nCRT and pCT patients. For more details, refer to Fig. 2 illustrating the Kaplan–Meier curves of the 1-year-survival.

**Table 2:** Univariate analysis comparing nCRT and pCT cohort after 1:1 propensity score matching

Variable	nCRT versus pCT (reference = nRCT)		
	*N* (nCRT/pCT)	HR (95%**—**CI)	*p* value
Clavien–Dindo score (Ref. = 0)	**44/31**		
1	2/3	2.13 (0.34–13.50)	0.423
2	8/9	1.60 (0.55–4.60)	0.386
3a	30/42	1.99 (1.03–3.83)	0.040
3b	7/5	1.01 (0.29–3.49)	0.983
4a	6/5	1.18 (0.33–4.22)	0.796
4b	0/2	N.A.	N.A.
5	0/0	N.A.	N.A.
Lymph nodes (Ref. = normal lymph nodes)			
Mean number of harvested lymph nodes per patient	28.86/35.81	(3.84–10.08)	< 0.001
Mean number of positive lymph nodes per patient	1.74/3.19	(0.20–2.69)	0.023
Anastomotic leakage (Ref. = no)	**91/86**		
Yes	6/11	2.00 (0.68–5.85)	0.206
Conduit necrosis (Ref. = no)	**97/95**		
Yes	0/2	N.A.	
Reintubation (Ref. = no)	**93/92**		
Yes	4/5	1.25 (0.34–4.7)	0.739
Rethoracotomy (Ref. = no)	**58/66**		
Yes	39/31	0.65 (0.34–1.25)	0.198
Tracheobronchial fistula (Ref. = no)	**97/91**		
Yes	0/1	N.A.	
Enterothorax (Ref. = no)	**97/97**		
Yes	0/0	N.A.	
Chylothorax (Ref. = no)	**95/96**		
Yes	2/1	N.A.	
Pylorospasm (Ref. = no)	**74/61**		
Yes	23/36	1.77 (0.97–3.20)	0.061
Pulmonal complications (Ref. = no)	**77/69**		
Yes	20/28	1.53 (0.80–2.94)	0.198
Cardiovascular complications (Ref. = no)	**89/91**		
Yes	8/6	0.71 (0.23–2.25)	0.566
Urinary tract infection (Ref. = no)	**97/97**		
Yes	0/0	N.A.	
Wound infections (Ref. = no)	**95/96**		
Yes	2/1	N.A.	
Catheter-associated infections (Ref. = no)	**89/89**		
Yes	8/8	1.00 (0.35–2.85)	> 0.999
Sepsis (Ref. = no)	**96/96**		
Yes	1/1	N.A.	
General infections (Ref. = no)	**94/94**		
Yes	3/3	N.A.	
Neurological complications (Ref. = no)	**93/93**		
Yes	4/4	1.00 (0.25–4.00)	> 0.999
Gastrointestinal infections (Ref. = no)	**96/97**		
Yes	1/0	N.A.	
Repeated stay on ICU (Ref. = no)	**93/93**		
Yes	4/4	1.00 (0.25–4.00)	> 0.999

*ICU* intensive care unit

*p* < 0.05 was considered statistically significant

We performed multivariate analysis and identified treatment with pCT, younger age, and Charlson score as independent variables for pylorospasm within the study cohort (see Table 3).

**Table 3:** Multivariate analysis comparing nCRT and pCT cohort after 1:1 propensity score

Variable		Pylorospasm	
	Yes/no	HR (95%—CI)	*p* value
Treatment (Ref. = nCRT); *N*	23/74		
Treatment pCT; *N*	36/61	2.41 (1.12–5.18)	0.024
Age; mean	60.8/62.6	0.84 (0.73–0.97)	0.018
BMI; mean	28.1/27.3	0.69 (0.45–1.08)	0.106
Charlson score; mean	3.97/3.93	3.59 (1.05–12.25)	0.041

		1-year survival	
	Deceased/alive	HR (95%—CI)	*p* value
Treatment (Ref. = nCRT); *N*	28/69		
Treatment pCT; *N*	26/71	1.08 (0.49–2.36)	0.848
Sex (Ref. = female); *N*	11/13		
Male	43/127	0.04 (0.00–1.12)	0.058
BMI; mean	27.2/27.7	0.68 (0.39–1.20)	0.182

## Discussion

It is widely accepted that patients benefit from multimodal therapy in locally advanced adenocarcinoma of the esophagus or gastroesophageal junction since their postsurgical prognosis is improved compared to patients only receiving oncological tumor resection. However, several different neoadjuvant (chemoradiation) such as the CROSS trial (Van Hagen et al. 2012) and perioperative (chemotherapeutic) treatment options as the FLOT regimen (Al-Batran et al. 2008) have been established and it is unknown which concept is superior so far. The question of the possible advantage of one therapy over the other is based not only on the corresponding tumor response but also on the perioperative mortality and the complications associated with the treatment itself. Both aspects must be evaluated carefully to conclude. We performed the current study, analyzing the therapeutic modalities of the CROSS and the FLOT regimen to address this question. Performing 1:1 propensity score matching for harmonization, we primarily focused on postoperative mortality as well as morbidity and secondly on histopathological tumor response after multimodal treatment followed by hybrid Ivor Lewis esophagectomy.

Most data concerning the histopathological response and especially the treatment-associated morbidity/mortality derived from retrospective analyses including a variety of different therapeutic regimens. To our best knowledge, there is no study available focusing exclusively on nCRT analogous CROSS versus pCT analogous FLOT. Only one recent analysis of our group addressed the subject so far: in the first retrospective approach, we compared a much smaller cohort of 40 propensity score-matched patients each after either nCRT or pCT (Favi et al. 2017). Contrary to the current study, the focus was on the postoperative histopathological response and prognosis of the patients, neglecting the important aspects of short-term postsurgical mortality/morbidity as well as the occurrence of postsurgical complications. In addition, the recruitment periods (2011–2015 versus 2013–2017) differ between the current and the previous assessment, so that the patient collective under consideration only partially overlapped. We could confirm our previous results and found no prognostic differences depending on the choice of multimodal treatment. Astonishingly and in contrast to our former study or data of other authors (Markar et al. 2017), we were not able to identify a better tumor response [major response: nCRT group (17/40 pts. 43%) versus pCT group (11/40 pts. 27%)] or a reduced rate of lymphatic metastasis (ypN0 = 68% versus ypN0 = 40%; *p* = 0.014) among patients after nCRT within the new, larger cohort of 97 propensity score-matched patients each (Favi et al. 2017). Surprisingly, before the propensity score matching, there was a higher rate of complete responders among nCRT patients when considering the absolute numbers (nCRT: ypN0 in 80/339 versus pCT: ypN0 in 13/97; *p* = 0.003). Similar findings were observed for the number of residual nodal metastasis in favor for nCRT (nCRT: ypN0 = 209/339 versus pCT: ypN0 = 40/97; *p* = 0.002). After matching, the number of retrieved lymph nodes per patient differed significantly between both groups with 28.86 lymph nodes within the nCRT group compared to 35.81 lymph nodes per patient within the pCT cohort (*p* < 0.001). These observations have also been made by Makar et al. (2017). Within their multicenter propensity score-based study including the results of multimodal treated patients with esophageal or junctional adenocarcinoma at ten European centers from 2001 to 2012, the authors compared the patients’ survival, short-term mortality, and morbidity as well as histopathological results of 301 patients in the nCRT and another 307 patients in the pCT group resulting in the largest single work considering this issue so far (Markar et al. 2017). Markar et al. described a larger number of harvested lymph nodes among pCT patients [27 (pCT) versus 14 (nCRT); *p* < 0.001] being associated with a lower rate of recurrence and an improved disease-free survival within this cohort of their study. Additionally, other authors reported a lower nodal retrieval among patients after nCRT compared to surgery alone (Talsma et al. 2014). Nevertheless, it is important to emphasize the differences within the study design of the current analysis: contrary to Markar et al. only patients who underwent Ivor Lewis esophagectomy were included neglecting those who received transhiatal gastrectomy or other procedures. Additionally, their study implemented several chemotherapeutic regimens such as MAGIC, OEO2 or OEO5 regimens (and not FLOT). Interestingly, the lymph node harvest was higher in our single-center study compared to the multicenter analysis. This might be due to the fact that we only included one highly standardized surgical technique instead of different technical approaches in several institutions. After all, both therapeutic strategies seem to provide comparable local regional control since (at least) the short-term postsurgical prognosis did not differ significantly. A recently published meta-analysis by van den Ende et al. (2020) draw a similar conclusion analyzing a total of 13 studies directly comparing nCRT analogous CROSS versus pCT analogous FLOT: none of these therapeutic options showed superiority considering the overall survival.

We chose the FLOT regimen excluding other chemotherapies since this protocol has demonstrated its efficacy and was superior compared to those other commonly used regimens such as ECX (Al-Batran et al. 2016, 2019). Nevertheless, several other studies analyzed the differences between nCRT and pCT others than FLOT. In 2019, Koch et al. published an analysis comparing the CROSS protocol with the EOX-protocol (epirubicin, oxaliplatin, xeloda) in patients with adenocarcinoma of the distal esophageal (GEJ I) (Koch et al. 2019). Considering the retrospective datasets of four Austrian centers between January 2007 and October 2017, they constructed a propensity score matching between 53 patients with nCRT and 51 patients with pCT. The authors described a significantly better local tumor control and better histopathological response after nCRT, which was similar to our findings in 2017 (Favi et al. 2017). However, the postsurgical prognosis was significantly improved in the pCT cohort (1-year survival rates: pCT = 92% versus nCRT = 85%) (Koch et al. 2019). In our current analysis, the postsurgical 1-year survival rates were not different in patients receiving CROSS and FLOT protocols. The same conclusion was reached by Markar et al. in their large European multicenter study comparing CROSS versus various perioperative chemotherapeutic regimens (Markar et al. 2017) although these colleagues included a longer postsurgical follow-up period presenting the patients’ 3-year survival.

An interesting aspect when discussing the choice of multimodal treatment regimens is the rate of complications derived from the neoadjuvant therapy itself. Since the current study focused on the short-term prognosis as well as the postsurgical complications on the one hand and the fact that most of the patients received their treatment elsewhere, we, unfortunately, were not able to implement this in our analysis. However, a recent Dutch series comparing patients after nCRT (CROSS) (*n* = 176) versus pCT (with different regimens such as MAGIC, ECX, EOX, or ECF) (*n* = 137) implemented such presurgical data (Anderegg et al. 2017). According to Anderegg and coworkers, the majority of nCRT patients underwent all previously planned treatment cycles (92.0%), while this was not true for around one-quarter of the pCT cohort (76.6%). Additionally, the localization, as well as the degree of manifestation of serious side effects and toxicities, differed: Esophagitis was predominant among nCRT patients whereas patients after pCT showed more often thromboembolic events, febrile neutropenia (associated with two presurgical deaths), nausea, vomiting, diarrhea, hand-foot syndrome, mucositis, cardiac complications or electrolyte imbalances (Anderegg et al. 2017). Therefore, the authors estimated the nCRT to be the preferred protocol in multimodal therapeutic concepts for esophageal or gastroesophageal adenocarcinoma as the expected severe adverse effects might be decreased compared to pCT.

As discussed before, the histopathological treatment response and the treatment-related toxicities are just a few aspects reflecting the superiority of a multimodal concept. Another important issue is the associated short-term morbidity/mortality as well as complications during the postsurgical course. In the recent past, several analyses were published covering this topic. These studies did not compare FLOT versus CROSS, but different regimens of pCT versus nCRT (Stahl et al. 2009; Swisher et al. 2010; Burmeister et al. 2011; Klevebro et al. 2016) in cohorts that were either exclusively patients with esophageal/gastroesophageal adenocarcinoma (Stahl et al. 2009; Burmeister et al. 2011) or where the majority of patients showed this histological subtype (Swisher et al. 2010; Klevebro et al. 2016; Nusrath et al. 2019). The results are still heterogeneous. Visser et al. (2018) published an Australian propensity score matching analysis considering 131 patients with EAC after nCRT and pCT each of who underwent surgery between 2000 and 2017. The authors compared the MAGIC-trial protocol with a radiation therapy including two cycles of cisplatin and 5-FU at a dose of either 35 Gy in 15 fractions or 45 Gy in 25 fractions. In the study’s last 2 years, the nCRT was changed to the CROSS protocol in the majority of patients (Visser et al. 2018). There were comparable rates of postoperative complications and in-hospital mortality in both cohorts. This is in concordance with our current study where we did not identify major differences in morbidity between patients after nCRT or pCT except for grade 3a complications according to the Clavien–Dindo classification. These complications were more frequent after pCT. The number of more serious complications was small in both cohorts and did not differ significantly. We reported an incidence of anastomotic leakage of 8.8% overall, with fewer in the nCRT cohort (*n* = 6, 6.3%) compared to the pCT cohort (*n* = 11, 11.3%). Anderegg et al. (2017) also observed non-significant differences considering anastomotic leakage within their retrospective study with incidences of 12.8% versus 19.1% (*p* = 0.134) while, in contrast to this, Markar et al. (2017) described within their propensity score-matched multicenter analysis a highly significant increased leakage rate among nCRT patients [23.1% (nCRT) versus 6.8% (pCT); *p* < 0.001]. Astonishingly, there was a trend towards a more frequent pylorospasm among pCT patients although this finding was non-significant. These observations might reflect the systemic (side) effects of the pCT in comparison to the more locally focused impact of the nCRT. Maybe the isolated increase of grade 3a complications was associated with the higher rate of pylorospasm in pCT since each postoperative endoscopy (for e.g., dilatation due to delayed gastric emptying) was considered as such a complication.

After all, the exact pathophysiological reason for this slightly higher rate of pylorospasm after pCT is still unknown. Independent from the kind of neoadjuvant therapy it is due to the radical nature of the surgery that a vagotomy is often inevitable and, therefore, the parasympathetic effects of the vegetative nervous system can no longer affect the function of the pylorus. Still today this is a common circumstance directly influencing the patients’ postsurgical gastrointestinal function and quality of life (Maus et al. 2016; Zhang and Zhang 2019; Yang et al. 2020). However, to our best knowledge, there are almost no data available so far focusing on this important issue. Sung et al. (2012) analyzed the effects of neoadjuvant chemotherapy on the neuro-muscular gastric function via examining stomach sections from patients with gastroesophageal adenocarcinoma in tissue baths for electrical field stimulation. Within their experimental setting, the authors compared three groups of patients: (1) Patients primarily undergoing (*n* = 3) surgery versus (2) patients who either received cisplatin and 5-fluorouracil (*n* = 2) due to esophageal cancer or (3) those who had epirubicin, cisplatin, and capecitabine (*n* = 2) due to the gastric adenocarcinoma. Carbachol-induced contraction of the isolated stomach tissue was significantly reduced after neoadjuvant chemotherapeutic treatment in both patients with gastric and esophageal cancer. Additional immunohistochemistry revealed decreased levels of acetylcholinesterase in both subgroups after chemotherapy (each *p* < 0.03) indicating that those agents can reduce the cholinergic function within the gastric neurotransmission (Sung et al. 2012). Interestingly, Sung et al. described an increase of ghrelin and motilin as putative mechanisms for compensation of the impaired gastric prokinetic activity after neoadjuvant chemotherapy. Nevertheless, these data just base on a very small number of patients included but might deliver useful hints about the possible pathomechanism we observed within the current study.

Following our data, Stahl et al. (2009), Burmeister et al. (2011), Klevebro et al. (2016), and Visser et al. (2018) did not identify significant differences in the postsurgical mortality as well as surgical/non-surgical complications after multimodal treatment between nCRT and pCT within their collectives. However, Burmeister et al. (2011) reported a higher rate of wound infections in the subgroup of patients after nCRT as a possible consequence of the applied radiation. In contrast to this observation, Swisher et al. postulated that surgical complications such as pulmonary insufficiency (*p* = 0.007), reintubation (*p* = 0.002), cardiovascular complications (such as arrhythmia; *p* = 0.012) or anastomotic leakages (*p* = 0.03) were significantly increased after nCRT while the surgical mortality was not affected by the choice of multimodal concept (Swisher et al. 2010). A possible explanation for this might be associated with the effects of irradiation on the lung parenchyma. Interestingly, Visser et al. (2018) demonstrated 90-day mortality of 2% in both nCRT and pCT, whereas the largest study focusing on this issue reported a 90-day mortality of 5.9% for nCRT and 2.3% for pCT (*p* = 0.090) (Markar et al. 2017). We did not observe this within our cohorts. All patients survived the first postsurgical 90 days. Nevertheless, it should be kept in mind that all these studies compared different chemotherapeutic and radiation regimens and recruited patients who underwent different surgical approaches not only Ivor Lewis esophagectomy, but also transhiatal gastrectomy or three-field esophagectomy. Additionally, as mentioned before, not all studies focused on EAC, but also ESCC.

Regarding these ambiguous data, the need for prospective randomized larger studies is evident. First prospective studies comparing nCRT versus pCT such as the ESOPEC (NCT02509286), Neo-AEGIS (NCT01726452) or TOGEAR study (NCT01924819) have been initiated within the recent past (Leong et al. 2015; Hoeppner et al. 2016; Reynolds et al. 2017). In detail, only ESOPEC focuses on the direct comparison between the FLOT and CROSS regimen for both distal adenocarcinomas of the esophagus as well as adenocarcinomas of the gastroesophageal junction (GEJ I–III) (Hoeppner et al. 2016). Its primary endpoint is the 3-year survival rate. Neo-AEGIS and TOGEAR also consider the possible differences in dependence of the respective neoadjuvant therapy (pCT versus nCRT) but do not include the exact protocols analogous to CROSS or FLOT (Leong et al. 2015; Reynolds et al. 2017).

On the one hand, the current study has its limitations due to its retrospective monocentric character. On the other hand, being performed in just one high-volume surgical single-center guaranteed a high degree of standardization (e.g., only including patients who underwent Ivor Lewis esophagectomy and received surgery in a standardized manner as well as the central and standardized data collection through the department’s outpatient clinic) improving the study’s data quality.

In conclusion, we demonstrated both nCRT analogous CROSS and pCT analogous FLOT to be safe and efficient within the multimodal treatment concept of patients with esophageal/gastroesophageal adenocarcinoma. Postsurgical mortality and complication rates were comparable and rare events. However, there were hints that functional aspects such as postoperative gastric emptying after pCT seem to be more frequently impaired.

## Data Availability

The datasets generated and/or analyzed during this current study are available from the corresponding author on reasonable request.

## Abbreviations

AIC: Akaike information criterion
EAC: Adenocarcinoma of the esophagus
ECCG: Esophagectomy Complication Consensus Group
GEJ: Gastroesophageal junction
nCRT: Neoadjuvant chemoradiation
pCT: Perioperative chemotherapy
